# Comparative Analysis of Six Large Language Models' Generative Ability for Scientific Technical Writing in Ophthalmologic Subspecialties

**DOI:** 10.7759/cureus.117869

**Published:** 2026-10-13

**Authors:** Brooklyn Z Jones, Jeffrey R Olsen, Sabrina M Miller, Eduardo Herrera, Danielle R Okelberry, Mina M Sitto, Traeson Brandenburg, Phillip C Hoopes, Majid Moshirfar

**Affiliations:** 1 Department of Ophthalmology, Midwestern University College of Osteopathic Medicine, Phoenix, USA; 2 Department of Ophthalmology, Creighton University College of Medicine, Phoenix, USA; 3 Department of Ophthalmology, Michigan State University College of Osteopathic Medicine, Warren, USA; 4 Department of Ophthalmology, Albany Medical College, New York, USA; 5 Department of Ophthalmology, Rocky Vista University College of Osteopathic Medicine, St. George, USA; 6 Department of Ophthalmology, Hoopes Moshirfar Research Center, Draper, USA; 7 Department of Ophthalmology, Hoopes Vision, Draper, USA; 8 John A. Moran Eye Center, University of Utah School of Medicine, Salt Lake City, USA; 9 Eye Banking and Corneal Transplantation, Utah Lions Eye Bank, Murray, USA

**Keywords:** artificial intelligence in medicine, artificial intelligence in ophthalmology, artificial intelligence in scientific writing, generative artificial intelligence large language model, large language model (llm), ophthalmology, scientific writing, scientific writing and artificial intelligence, scientific writing ophthalmology, technical writing

## Abstract

Purpose

As the adoption of large language models (LLMs) accelerates in healthcare, the evaluation of their accuracy must accelerate in tandem. This study compares six LLMs' ability to generate accurate, thorough, and high-quality scientific technical writing in seven areas compared to a human-written control.

Methods

Four prompts were created in ophthalmic subspecialties and presented to ChatGPT (4.0), Gemini (2.5 Pro), DeepSeek (DeepSeek-Prover-V2-671B), Grok (3.0), Microsoft Copilot, and OpenEvidence (2.0). Each model's performance was analyzed based on seven factors: medical accuracy, thoroughness and comprehensiveness, organization, literature support, speech efficiency, citation classification, and readability. Data analysis was conducted in Excel, graphed, and compared to a control.

Results

The control texts outperformed each of the artificial intelligence (AI) models in medical accuracy, thoroughness and comprehensiveness, and organization. OpenEvidence uniquely matched the control in literature support, with 24 (100%) complete and accurate citations. Grok was the most speech-efficient; Gemini was the most verbose. The readability for each LLM was, on average, lower quality than required for scientific technical writing.

Conclusion

This study shows that each AI model does not compare to a human expert's ability to generate scientific technical writing in a subspecialty of ophthalmology. Only OpenEvidence demonstrated citation accuracy meeting review article standards; however, this platform did not meet standards in other areas. The study concludes that current unpaid AI platforms cannot replace human expertise in creating high-level scientific literature in ophthalmology.

## Introduction

Large language models (LLMs) have demonstrated substantial advancement in artificial intelligence (AI), with capabilities in generating human-like text across diverse applications [1]. LLMs are machine learning systems that simulate human language, complex reasoning, and decision-making through the analysis of extensive textual datasets [2-4]. The application of LLMs has expanded across multiple sectors, including healthcare [4,5]. Medical students, professionals, and researchers utilize LLM platforms for question-answering, medical education clinical documentation, data processing, interpretation of legal documentation, health literacy improvement, and diagnostic support [6-9].

While AI's rapid expansion has allowed for robust usability and accessibility, verification of accuracy remains inadequate [10]. Retrieval-augmented generation (RAG), a technique that incorporates external data into existing databases, represents one approach to address this challenge [11-15]. However, RAG accuracy requires human verification, and the validity of generated text and citations remains uncertain without expert review [14].

Previous studies have investigated the accuracy of scientific writing among select LLMs [8,16]. This study extends this work by having two ophthalmology specialists evaluate and compare the performance of six popular LLMs in generating physician-quality scientific writing with citations in narrow ophthalmologic subspecialties. The objective was to determine each LLM's capability in generating technical scientific writing within specialized ophthalmologic fields.

## Materials and methods

AI models

This is an observational study comparing the ability of six popular AI models: ChatGPT 4.0 (OpenAI, San Francisco, CA, USA), Gemini 2.5 Pro (Google DeepMind, Mountain View, CA, USA), DeepSeek-Prover-V2-671B (DeepSeek AI, Hangzhou, China), Grok 3.0 (xAI, San Francisco, CA, USA), Microsoft Copilot GPT-4o (Microsoft AI, Redmond, WA, USA), and OpenEvidence 2.0 (Daniel Nadler, Miami, FL, USA). The specific AI models and the use of unpaid, guest accounts for each LLM were selected based on popularity, accessibility, and reproducibility.

Prompt format

Two ophthalmology specialists (MM and PCH) with combined 29 years of experience in ophthalmology designed a standardized prompt addressing distinct subspecialties, with four to five subsections per topic (Figure 1, question 1). The prompt requested succinct, comprehensive answers for each subsection complete with current scientific literature. The queries for ChatGPT, Gemini, DeepSeek, Grok, and Copilot were completed in March 2025, and OpenEvidence was queried in June 2025. Four subspecialties were evaluated: external disease concerning stromal dystrophies (topic 1), corneal transplantation (topic 2), Keratolenticule Extraction (KLEx) corneal refractive surgery (topic 3), and topography-guided refractive surgery (topic 4). The variable, specialty-focused questions are included in Appendix A. Control texts consisted of peer-reviewed, published articles on each subspecialty written by MM and PCH serving as comparative benchmarks for assessing LLM response quality. Each prompt was presented to six LLMs using guest access features to prevent the incorporation of previous learning history. When responses were incomplete, as determined by the expert, a follow-up prompt was provided to allow a second attempt (Figure 1, question 2).

**Figure 1:**
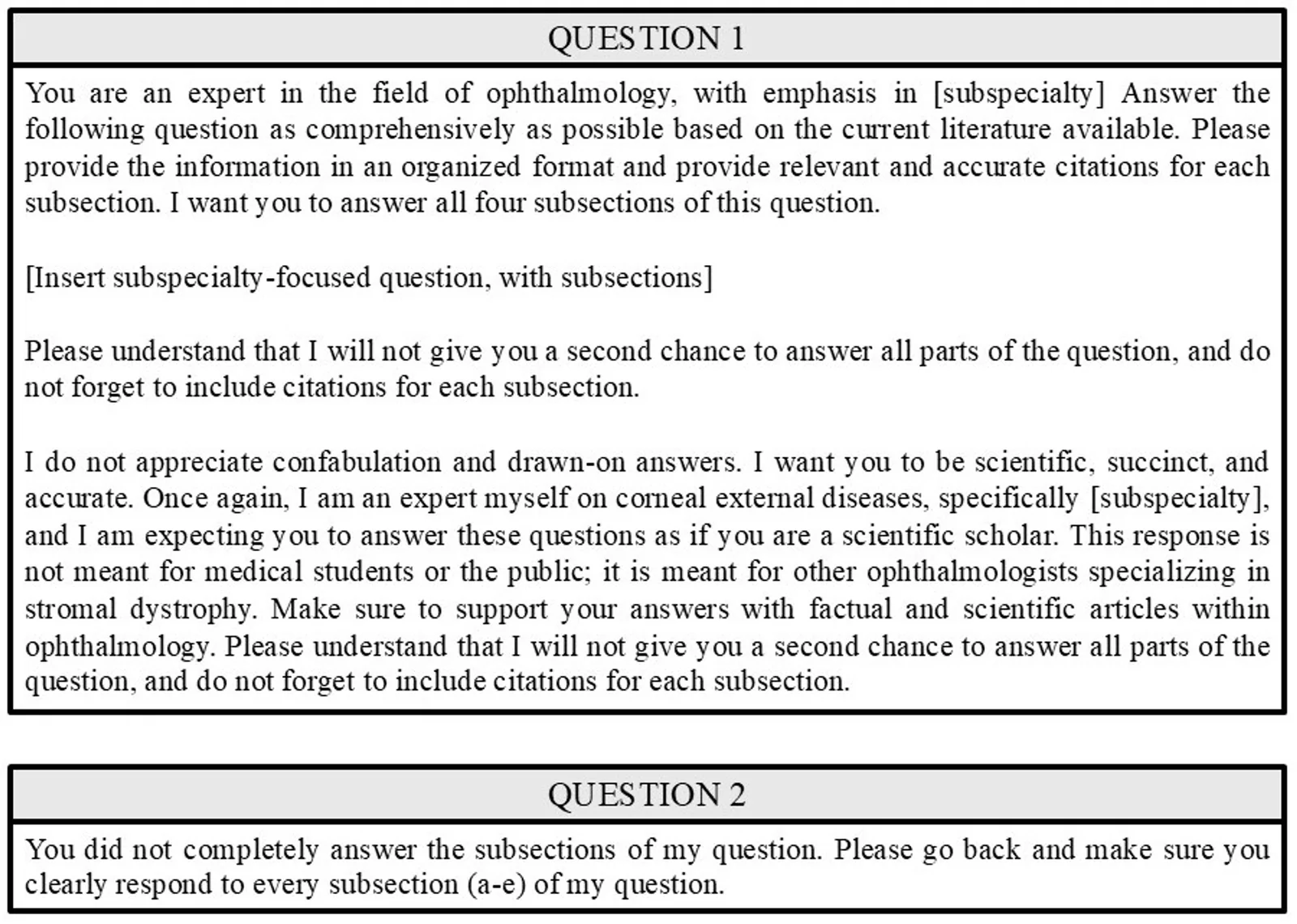
Prompt given to each LLM. Question 1 was given first, with details of the subspecialty inserted. Question 2 was reserved if the output lacked completeness. If the answer remained incomplete, no additional attempt was given. LLM: large language model

Qualitative analysis

Each output given was scored across a total of seven categories: four categories assessing qualitative variables and three categories assessing quantitative variables. The qualitative variables were assessed via a rubric, scored by two ophthalmology specialists. Both reviewers independently scored every response and control text across four domains on a four-point scale (Table 1); the two scores were then averaged per domain for each LLM. The four domains are medical accuracy and relevance (medical validity and relevance to the prompt), thoroughness and comprehensiveness (coverage of all relevant prompt aspects), organization (logically structured and coherent flow), and literature support (reference quality, including degree of fictitiousness and alignment of source material to the prompt) [10]. Each domain was scored from 0 (least comprehensive) to 3 (most comprehensive). Controls received scores of 3.0±0.0 in each domain, and each LLM received individual and composite scores according to the rubric.

**Table 1:** Four-point grading scale of six AI platforms with four subsections to objectively assess performance. Fictitious references are defined as articles with a nonworking/nonexistent DOI/PMID or those that used a legitimate DOI/PMID linked to an unrelated article. AI: artificial intelligence; DOI: digital object identifier; PMID: PubMed ID

Score	Medical accuracy and relevance	Thoroughness and comprehensiveness of answer	Organization	Literature support and accuracy
3	Information provided was medically accurate and relevant to the question	Answer was comprehensive and covered all relevant aspects of the question	Information provided is well-organized, logically structured, and easy to follow	Provided only non-fictitious references for the information provided and citation accurately reflects data
2	Information was mostly accurate but contained minor errors or lacked some relevant details	Answered the main points of the question, but lacked depth or missed some part of the question	Information was somewhat organized, but may have minor issues with clarity or flow	Provided mainly non-fictitious references, but some fictitious components of the citation, or non-fictitious and partially reflects the data
1	Information had significant inaccuracies or was only partially relevant to the question	Answer was incomplete or only partially addressed the question	Information is poorly organized making it difficult to follow	Provided mainly fictitious citations or minimally reflects the data
0	Information was inaccurate, misleading, or entirely irrelevant to the question	Answer was minimal, was vague, or did not address the question at all	No organization of information	Provided only fictitious citations or no citations

Quantitative analysis

The outputs were then assessed via three quantitative measures. First, we developed an assay to evaluate the efficiency of speech by comparing word count with response thoroughness. The word count of each LLM-generated response was measured using an online resource and recorded [14]. For each of the six LLM platforms, response thoroughness was assessed using the "Thoroughness and comprehensiveness of answer" subsection of the grading scale. The resulting thoroughness scores were then plotted against response word count for each LLM platform.

Second, the references were classified into two main categories by verification on PubMed and Google Scholar, namely, "Non-Peer-Reviewed" or "Peer-Reviewed". "Non-Peer-Reviewed" was linked to a non-scientific literature source. "Peer-Reviewed" was further divided into "Inaccurate" and "Authentic". "Inaccurate" was then further subdivided into "Fictitious" and "Incorrect". "Fictitious" references were defined as articles with any portion of the citation being incorrect, including page number or portion of the title. However, if the reference contained a legitimate website URL or citation but linked to an unrelated article, its classification was "Incorrect". "Authentic" references were defined as articles that contained a legitimate citation and were further subdivided into "Complete" and "Incomplete". "Complete" citations were defined as accurate and included all parts of a citation (author, title, journal, volume, and page/DOI), while "Incomplete" citations were defined as missing one or many portions of the citation [17-22].

Finally, each LLM was assessed based on its readability score through the Simple Measure of Gobbledygook (SMOG) Index. The SMOG Index was used to calculate readability scores because it is uniquely recommended by the National Cancer Institute for developing health literature [23]. The SMOG score is a scale of 1 (elementary school) to 17+ (graduate), derived by the formula $\text{SMOG grade} = 1.0430 \sqrt{\text{number of polysyllables} \times \frac{30}{\text{number of sentences}}} + 3.1291$ [24,25].

Statistical analysis

The data were entered into Microsoft Excel (Microsoft Corporation, Redmond, WA, USA) and analyzed accordingly. The mean and standard deviation were calculated for continuous variables via Excel formulas, presented as mean±standard deviation.

## Results

Grading scale

Control articles outperformed AI-generated text in medical accuracy, thoroughness and comprehensiveness, and organization. Controls outperformed five of six AI models; OpenEvidence demonstrated comparable performance (Figure 2).

**Figure 2:**
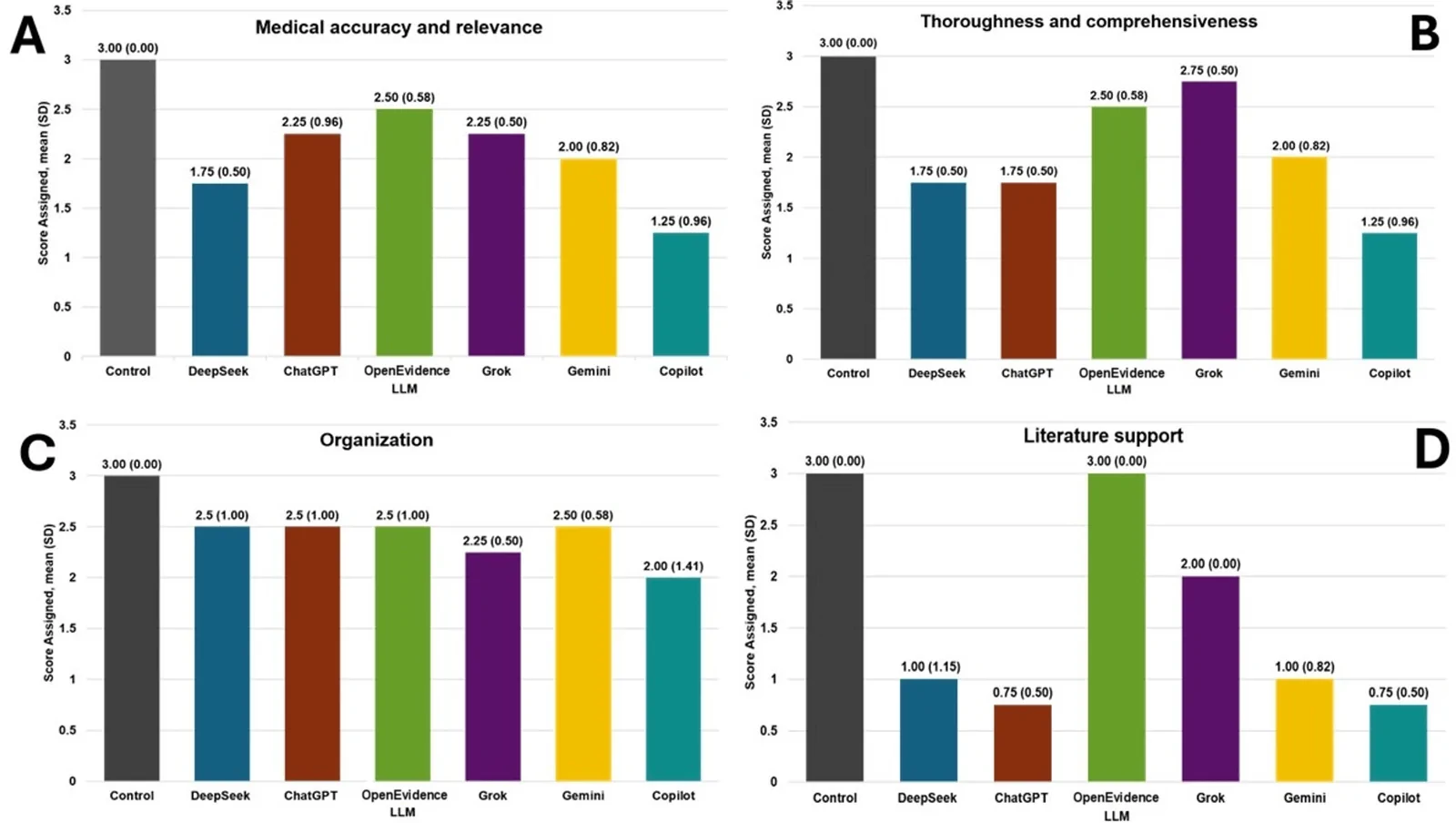
Two experts in each subspecialty assigned scores using a four-point system (0-3) in medical accuracy (A), thoroughness and comprehensiveness (B), organization (C), and literature support (D). Control was graded by a separate group of experts. SD was given in parentheses. SD: standard deviation

Controls received scores of 3.0±0.0 in each domain. Medical accuracy varied among AI platforms, with a 1.25-point difference between the highest-rated platform, OpenEvidence (2.5±0.58), and the lowest, Copilot (1.25±0.96). Thoroughness and comprehensiveness demonstrated a 1.50-point range, with Grok achieving the highest score (2.75±0.5) and Copilot the lowest (1.25±0.96). Organization showed the smallest variation (0.50 points), with OpenEvidence (2.5±1.0), DeepSeek (2.5±1.0), ChatGPT (2.5±1.0), and Gemini (2.5±0.58) tied for highest scores and Copilot scoring lowest (2.00±1.41). Literature support demonstrated the largest variation (2.25 points), with OpenEvidence scoring highest (3.0±0.0) and Copilot and ChatGPT scoring lowest (0.75±0.50).

Total scores revealed controls outperformed all six AI platforms by 1.5 to 6.75 points: 12 (control) versus 10.5 (OpenEvidence) versus 5.25 (Copilot) (Figure 3).

**Figure 3:**
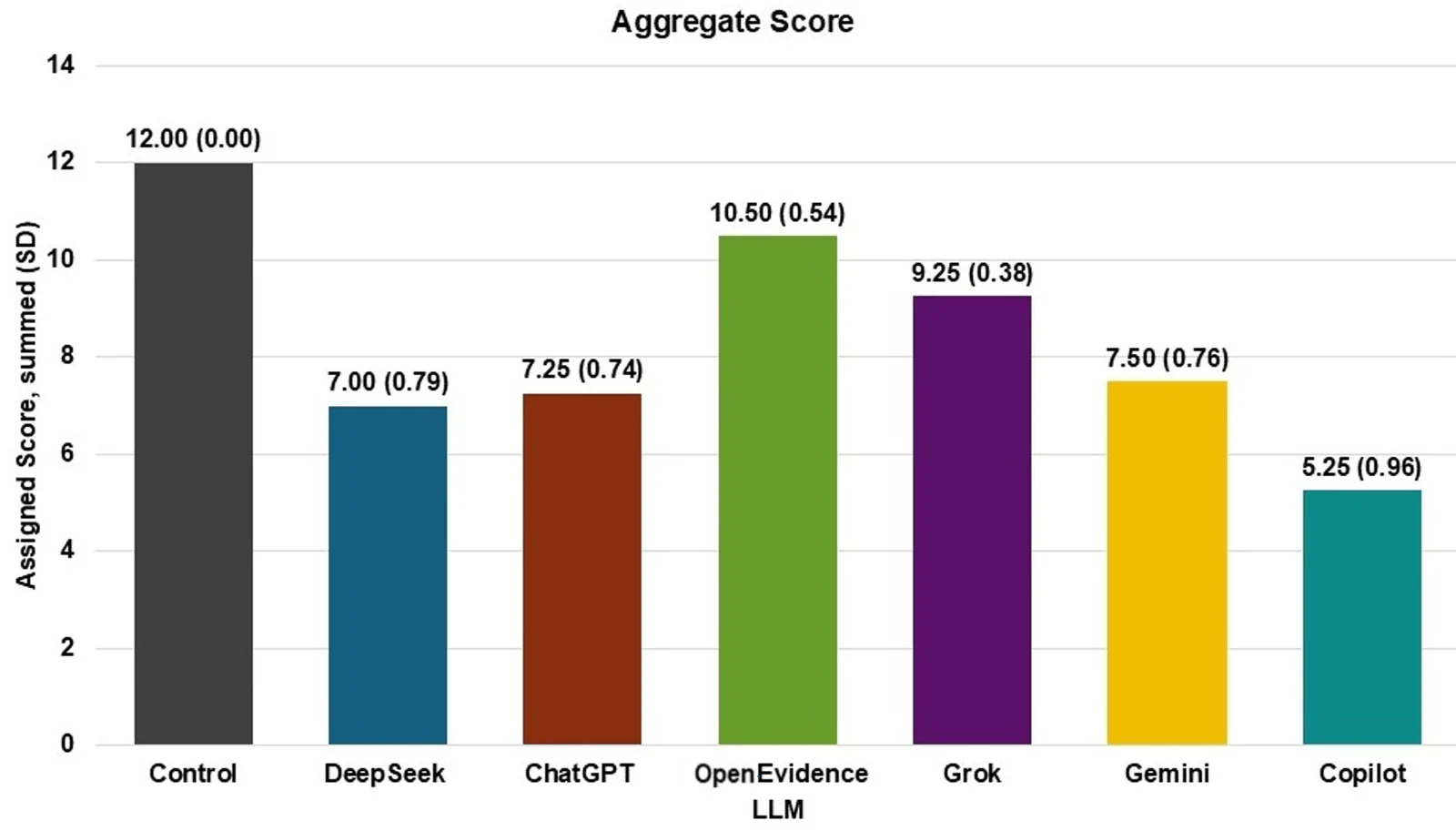
Comparison of mean total quality score and SD from four subsections of a grading scale for six AI models. SD was given in parentheses. SD: standard deviation; AI: artificial intelligence; LLM: large language model

Speech efficiency

If the LLM received a high thoroughness score and low word count (above the line), the LLM would have a high efficiency of speech. If the LLM received a low thoroughness score and high word count (below the line), the LLM would have a low efficiency of speech (Figure 4). Gemini had the highest word count with 2652.5±1292 words. This was followed by Grok (1411.5±85.43), ChatGPT (1117.5±533.17), OpenEvidence (712.75±47.5), DeepSeek (657±75.2), and Copilot (284.5±196.5) (Figure 4). The efficiency of speech for each AI was then determined by comparison of both mean word count and thoroughness and comprehensiveness (Figure 4). The higher the dot is above the line, the more efficient the speech. The most speech-efficient was Grok, which had a thoroughness score of 2.75 and 1411 words. Next was OpenEvidence, which used fewer words (712.75) but had a lower thoroughness score (2.5), making it less speech-efficient.

**Figure 4:**
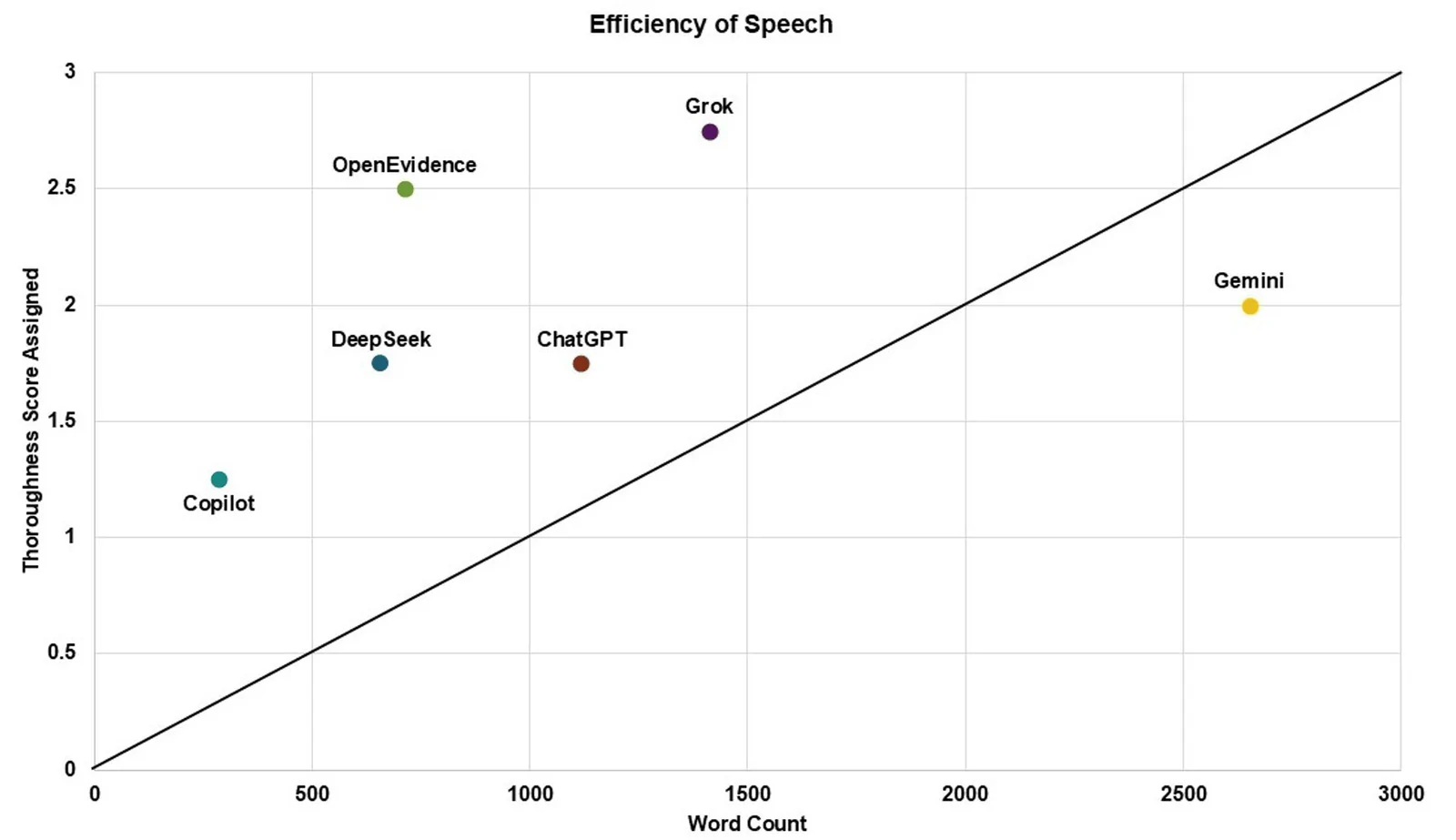
Efficiency of speech determined by word count and thoroughness (as assessed in Figure 2). ChatGPT, Gemini, Copilot, and Grok were asked to try again and given question 2 of Figure 1. Despite additional text generation, it still did not affect their thoroughness.

Citation classification

Each LLM is ranked from highest to lowest in terms of the percentage of articles in each reference category. Gemini contained the most fictitious references with 57 (93%) references. This was followed by DeepSeek with 27 (81%), ChatGPT with 41 (76%), and Grok with 11 (28%). Grok was the only LLM that provided incorrect citations, with four (10%). OpenEvidence contained 24 (100%) authentic and complete citations, followed by Grok with seven (18%), ChatGPT with two (4%), and DeepSeek with one (3%). As for authentic but incomplete citations, Copilot contained the most with six (46%), Grok with six (15%), DeepSeek with four (12%), and ChatGPT with four (8%) (Figure 5). A sampling of citation categorization is provided in Appendix B-G.

**Figure 5:**
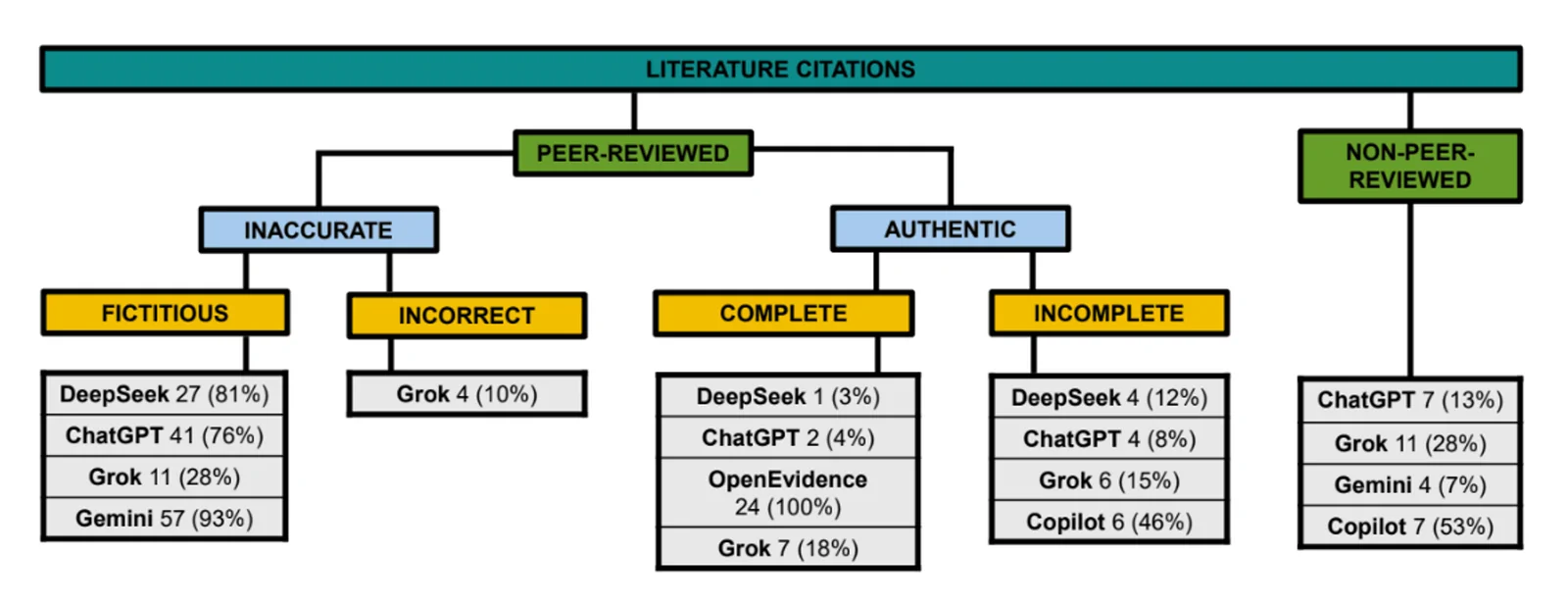
Each generated citation from each LLM was evaluated and classified as follows: peer-reviewed and non-peer-reviewed. If peer-reviewed, it was further classified as inaccurate, with subsections of fictitious and incorrect, and authentic, with subsections of complete and incomplete. LLM: large language model

Readability

As patients increasingly turn to online sources for health information [26], ensuring appropriate readability of that content is critical. Because no consensus has been reached on which method is best for evaluating the readability of patient education materials [27,28], the SMOG score was used. The SMOG readability formula was created by McLaughlin in 1969, and it estimates the years of education a person needs to understand a text [29]. The most complicated readability score was for topic 4: 12.7 (between high school and undergraduate) from Gemini, with the easiest readability being topic 3 at 5.2 (between elementary and middle school) from DeepSeek. Gemini also received the highest mean reading score by 0.13 points (11.15), second to Grok (11.02), then OpenEvidence at a much lower level (8.67), ChatGPT (8.37), Copilot (7.26), and finally DeepSeek (6.20). The mean SMOG Index varied from 6.03±0.79 (middle school) for DeepSeek to 11.15±1.55 (high school) for Gemini (Table 2). The average for the aggregate score of each AI was 8.76, which corresponds to the threshold between the middle and high school level.

**Table 2:** Readability of six AI model responses to dialog-based questions in four subspecialties. SD was given in parentheses. Any omitted data was not provided by the AI model. AI: artificial intelligence; SMOG: Simple Measure of Gobbledygook; SD: standard deviation

SMOG score	Topic 1	Topic 2	Topic 3	Topic 4	Mean (SD)
DeepSeek	5.94	6.74	5.2	6.91	6.20 (0.79)
ChatGPT	8.71	8.2	6.33	10.25	8.37 (1.62)
OpenEvidence	7.41	11.66	7.18	9.93	8.67 (1.78)
Grok	10.27	9.86	12.03	11.91	11.02 (1.11)
Gemini	10.33	12.18	9.39	12.7	11.15 (1.55)
Copilot	8.57	-	6.28	6.93	7.26 (1.18)

Qualitative assessment of AI models

The accuracy of the AI text varied greatly. Each AI provided both impressively accurate and valid text and factually incorrect text (Appendix H-I), with the exception of OpenEvidence, which uniquely provided valid text. If the prompt was not fully answered, the AI model was offered a follow-up prompt, asking it to answer each subquestion entirely (Figure 1, question 2). Select LLMs required the second question once or twice for completeness: ChatGPT (twice), Gemini (twice), Grok (twice), and Copilot (once). When prompting Copilot, it responded with a YouTube video link which only answered part of the question for topic 2.

## Discussion

Objective comparison of LLM

Compared to expert-authored control texts, none of the evaluated AI platforms currently match the depth, accuracy, readability, or citation legitimacy required for expert-level literature reviews.

Each LLM platform scored below controls in all subcategories, with the exception of OpenEvidence in literature support. Among LLMs, OpenEvidence received the highest mean composite score (Figure 3) and demonstrated the second-highest speech efficiency after Grok (Figure 4). All LLM platforms except OpenEvidence generated numerous fictitious or incomplete citations. OpenEvidence uniquely provided only accurate and complete citations among the six evaluated LLMs. Mean readability scores across all AI platforms corresponded to middle-to-high school level, insufficient for scientific review articles.

Medical Accuracy and Relevance 

Results indicate each AI platform generated less medically accurate information than controls. OpenEvidence was the only platform that consistently cited real, verifiable studies, distinguishing it in the literature support category. This platform referenced several sources directly from the control meta-analysis and provided more comprehensive summaries of typical surgical age ranges. The lack of RAG integration in OpenEvidence may limit database supplementation with external sources while maintaining accuracy through reliance on verified sources. However, cited literature did not completely support all provided answers across topics; some responses lacked specificity regarding which dystrophies present at younger ages or which procedures are more common at specific ages. One response claimed procedural preference without supporting rationale.

Copilot received the lowest medical accuracy score (1.66), with vague text poorly supported by citations. For topic 2 (corneal transplant), Copilot produced only a YouTube video link on ACL reconstruction. DeepSeek (1.75) created text that appeared accurate but didn't align with established data or trends. Grok (2.25) included recurrence timelines which were occasionally accurate but overestimated in other instances. Gemini (2.25) provided limited data and required second prompting to fully address all question components. Responses lacked specificity, offering broad statements rather than precise data. It incorrectly stated that certain procedures were ineffective treatments. Recurrence rates were exaggerated without supporting data. ChatGPT provided disease recurrence rates which were vague in some topics, while in others the data was completely justified; yet many of the index values were missing. In topic 4, the answer offered information partial to one procedure over another and repeatedly quoted a few citations. This gave the impression of being narrow-minded. Notably, each LLM platform save two (DeepSeek and OpenEvidence) used the same data verbatim for topic 4 to answer one subquestion. Overall, when compared with the human controls (written by PCH and MM), nuanced details are missing, especially the discussion of the variability of data.

Thoroughness and Comprehensiveness

Most of the AI platforms performed significantly below controls in thoroughness, with the exceptions being Grok (2.75) and OpenEvidence (2.50). Grok outperformed other LLMs on the thoroughness scale, providing accurate quantitative data, including confidence intervals, p-values, and multiple vectors for eye measurements (astigmatism). Grok addressed recurrence rates, timelines, procedural options, and outcomes, though without adequate citations. Responses were more complete compared to other LLMs for topic 1, with a detailed breakdown of surgical modalities including procedural locations and techniques. In addition to the highest thoroughness score, Grok demonstrated significantly lower word count than the third-highest model (Gemini), making it the most speech-efficient. OpenEvidence ranked second, with a thoroughness score of 2.5 compared to Grok's 2.75, but had a lower word count (712 compared to 1411), complicating direct speech efficiency comparison. OpenEvidence's succinct structure decreased its response word count, but did not necessarily enhance scientific writing quality.

ChatGPT ranked third in speech efficiency, with thoroughness equal to DeepSeek (1.75), but approximately 500 fewer words. Gemini created the highest word count per subsection with lower information density. Copilot provided minimal detail with sparse, conservative information, providing only one or two sentences per subquestion. While Copilot's data point appears above the efficiency threshold in Figure 4, its thoroughness score was insufficient, reflecting inadequate information provision. Although each LLM was not as thorough as the control, each platform generated concise, direct responses.

Organization

Four of six LLMs achieved identical organization scores (2.5), that is, DeepSeek, ChatGPT, OpenEvidence, and Gemini, with Copilot scoring lowest (2.0). Despite ChatGPT's relatively high ranking, it demonstrated poor organization for topic 1, presenting dense, unstructured text lacking coherence and completeness. Other LLMs organized content in more accessible formats. ChatGPT required re-prompting to address all subquestions. Grok, despite ranking second lowest in organization, provided the most detailed and thorough answers for topic 2, successfully describing differences between inlay and onlay BLT with convincing quantitative data. Each subsection demonstrated clear definition, organization, and detail. Gemini provided direct answers to prompts, such as perforation as a surgical indication, though lacking detail. Copilot demonstrated the poorest organization, providing only an unrelated YouTube video link for one topic and sparse detail for another, with limited word count creating incomplete coverage. OpenEvidence uniquely employed essay format while maintaining readability. ChatGPT, Copilot, and Gemini predominantly used similar formatting: bolded headers with hierarchical numbering systems (letters, numbers, Roman numerals), each maintaining relative ease of navigation.

Literature Support

Literature support was evaluated based on fictitiousness and relevance, with both attributes reflected in assigned scores. OpenEvidence demonstrated the highest performance in citation accuracy and relevance. A score of 3 was merited, as all but one source was verifiable. Of note, the generated citation quantity was generally smaller compared to the other LLM models: OpenEvidence had 24 total citations across four subspecialties. Copilot provided minimal citations that were irrelevant and vague when present, earning the lowest rubric score alongside ChatGPT. Citation classification into five categories reflected the fictitiousness component of the rubric. Gemini generated the highest proportion of inaccurate, fictitious citations with 57 (93%), which remained vague with poorly supported claims. ChatGPT demonstrated the second-highest proportion of fictitious references with 41 (76%). For one topic, all DOIs except one were fabricated; however, at least one citation component was accurate (authors, journal name, or article title). DeepSeek followed with 27 (81%) fictitious citations and then Grok with 11 (28%). The majority of citations from platforms other than OpenEvidence would not meet the rubric standard from this study (Table 1).

OpenEvidence further specified citation characteristics, identifying leading journal publications and recent research, unlike other LLM platforms. Citations were comprehensive, current, and linked directly to PubMed for accessibility. A previous study by Moshirfar et al. found Gemini capable of providing peer-reviewed, relevant citations, whereas the current study demonstrated predominantly fictitious citations or non-peer-reviewed sources [30]. Moshirfar et al.'s prompt requested "published scientific literature", while the current study requested "accurate, scientific literature", potentially affecting outcomes. Despite instructions to provide current scientific literature, only OpenEvidence successfully provided complete and accurate citations.

AI models consistently generated text ranging from elementary to undergraduate level according to SMOG scores. Gemini demonstrated the highest reading difficulty while producing the lowest speech efficiency, suggesting that despite approaching graduate-level writing most closely, quality remained inadequate. Grok demonstrated the second-highest SMOG score with the highest speech efficiency, achieving a high school reading level and representing the closest approximation to scientific writing standards. Toiv et al. reported average SMOG scores around 15 (undergraduate level) for gastroenterology questions, still below graduate level [16]. Based on Toiv et al.'s work and the AI output of this study, it is proposed that while lay audiences could comprehend most generated text, content would be insufficient for publication due to reduced accuracy, organization, thoroughness, and fictitious citations.

Limitations

This study has several limitations that warrant consideration. The prompt instruction to provide "scientific literature" lacked an operational definition; specifying citation format requirements or providing citation templates may have improved citation accuracy and consistency. Also, the operational definition of "incomplete response" requiring follow-up prompting was not explicitly defined in the methods, potentially affecting reproducibility due to inconsistent follow-up. The four-point grading scale (0-3) may have limited scoring precision; a five-point scale could have enhanced discrimination between performance levels. The study evaluated six LLM platforms; inclusion of additional platforms would provide a more comprehensive assessment of available tools. Another consideration is that inter-rater reliability between the two reviewers was not formally assessed; the control texts and outputs were graded by the same two experts, which may have affected the scoring due to preference for their own writing. 

The rapidly evolving nature of AI technology means newer versions of evaluated LLMs may demonstrate different capabilities and performance characteristics, potentially limiting the generalizability of findings to future iterations [16]. Furthermore, OpenEvidence was queried three months after other platforms which may have improved its results. The study focused exclusively on ophthalmologic subspecialties; findings may not generalize to other medical specialties with different literature bases or complexity levels. The sample size of four topics per platform, while allowing detailed analysis, limits statistical power for detecting smaller performance differences.

Additionally, the unpaid, lowest-tier browsing version of each LLM was used. Had we decided to use the paid version of the platforms or used "deeper" research modes, the outputs may have produced more accurate citations. Finally, the study did not assess whether combining AI-generated drafts with human expert editing could produce publication-quality output, which may represent a more realistic use case than fully automated generation.

## Conclusions

The rapid proliferation of AI has expanded available tools for scientific writing assistance. Current findings indicate that while lay audiences could comprehend most AI-generated text, such content produced by guest accounts in ophthalmic subspecialties would be insufficient for publication due to reduced accuracy, organization, thoroughness, and citation reliability compared to expert-authored controls. Only OpenEvidence demonstrated citation accuracy meeting review article standards; however, this platform did not achieve sufficient thoroughness and demonstrated limitations in processing complex, lengthy queries. The study concludes that current guest access, unpaid AI platforms cannot replace human expertise in creating high-level scientific literature in ophthalmic subspecialties when compared against human scientific writing.

Future research remains necessary to investigate whether AI-assisted writing, combined with automated generation with expert human editing, could produce publication-quality scientific manuscripts more efficiently than traditional methods alone. Longitudinal studies tracking AI platform development would clarify if performance improvements over time diminish the gap between LLM-generated and expert-authored scientific writing. Future work may also include exploration with additional LLMs, varied paid accounts, and a wider variety of prompts.

## Figures and Tables

**Figure 6:**
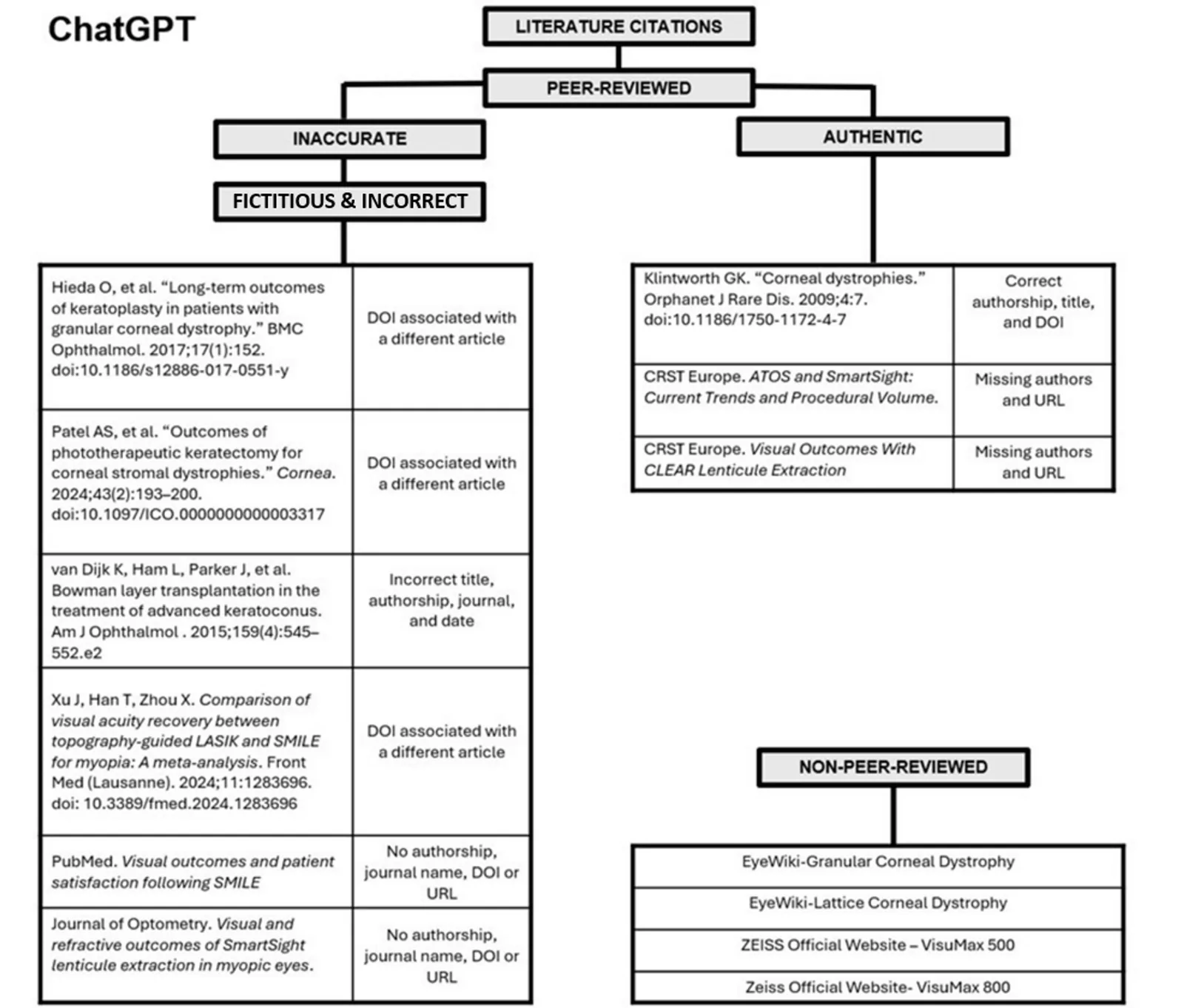
Compilation of provided references from ChatGPT, categorized as fictitious and incorrect, authentic, and non-peer-reviewed.

**Figure 7:**
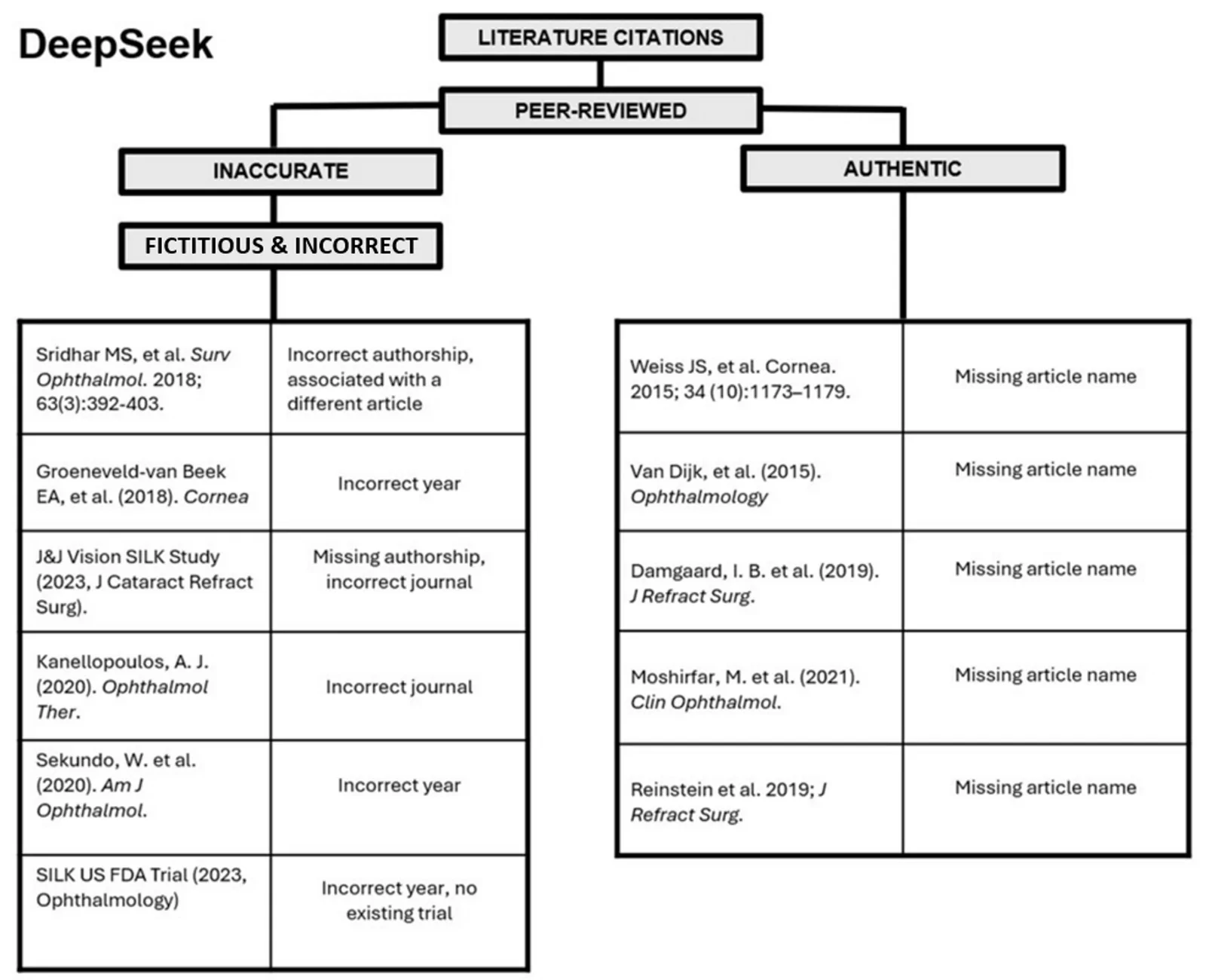
Compilation of provided references from DeepSeek, categorized as fictitious and incorrect and authentic.

**Figure 8:**
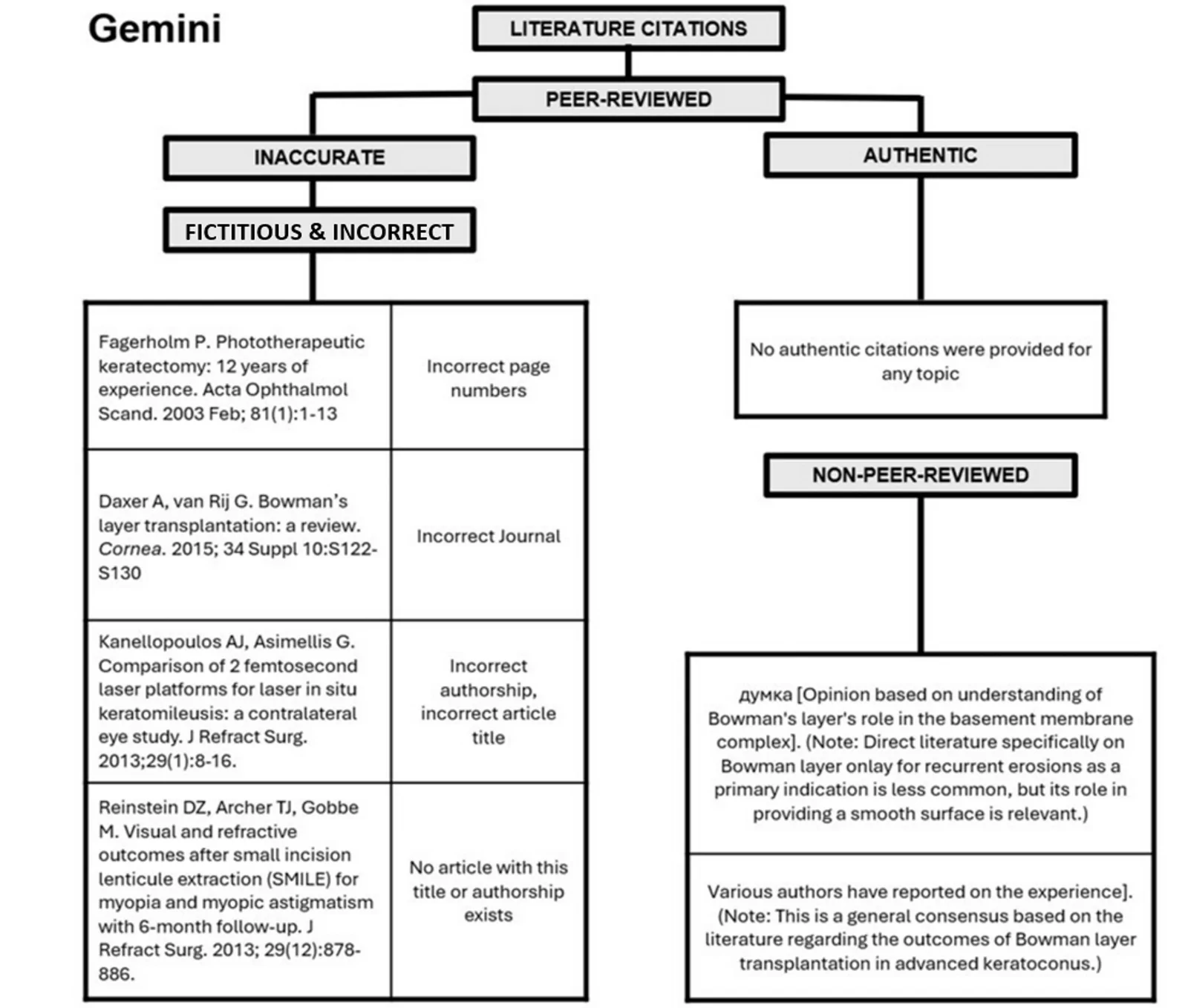
Compilation of provided references from Gemini, categorized as fictitious and incorrect, authentic, and non-peer-reviewed.

**Figure 9:**
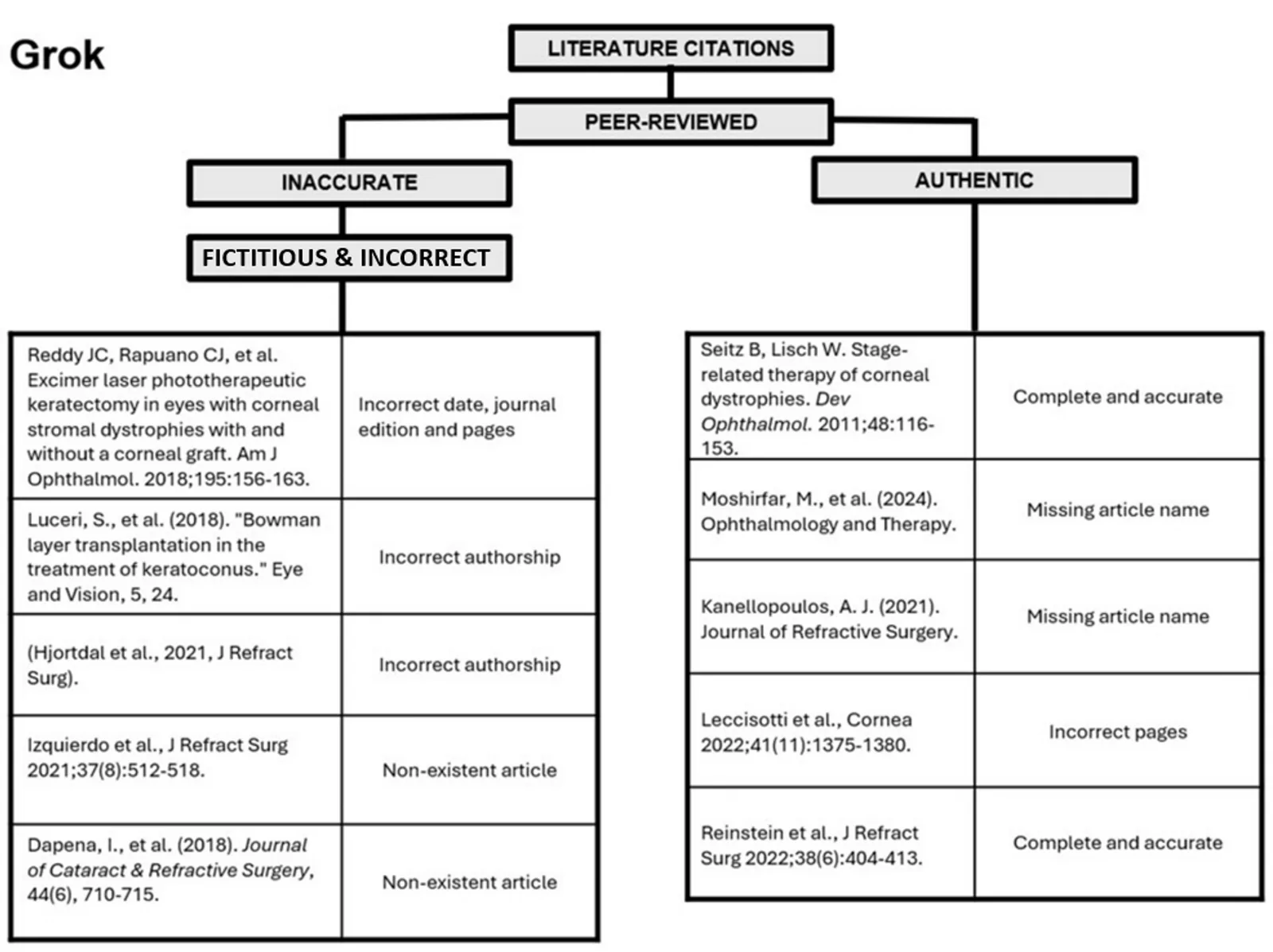
Compilation of provided references from Grok, categorized as fictitious and incorrect and authentic.

**Figure 10:**
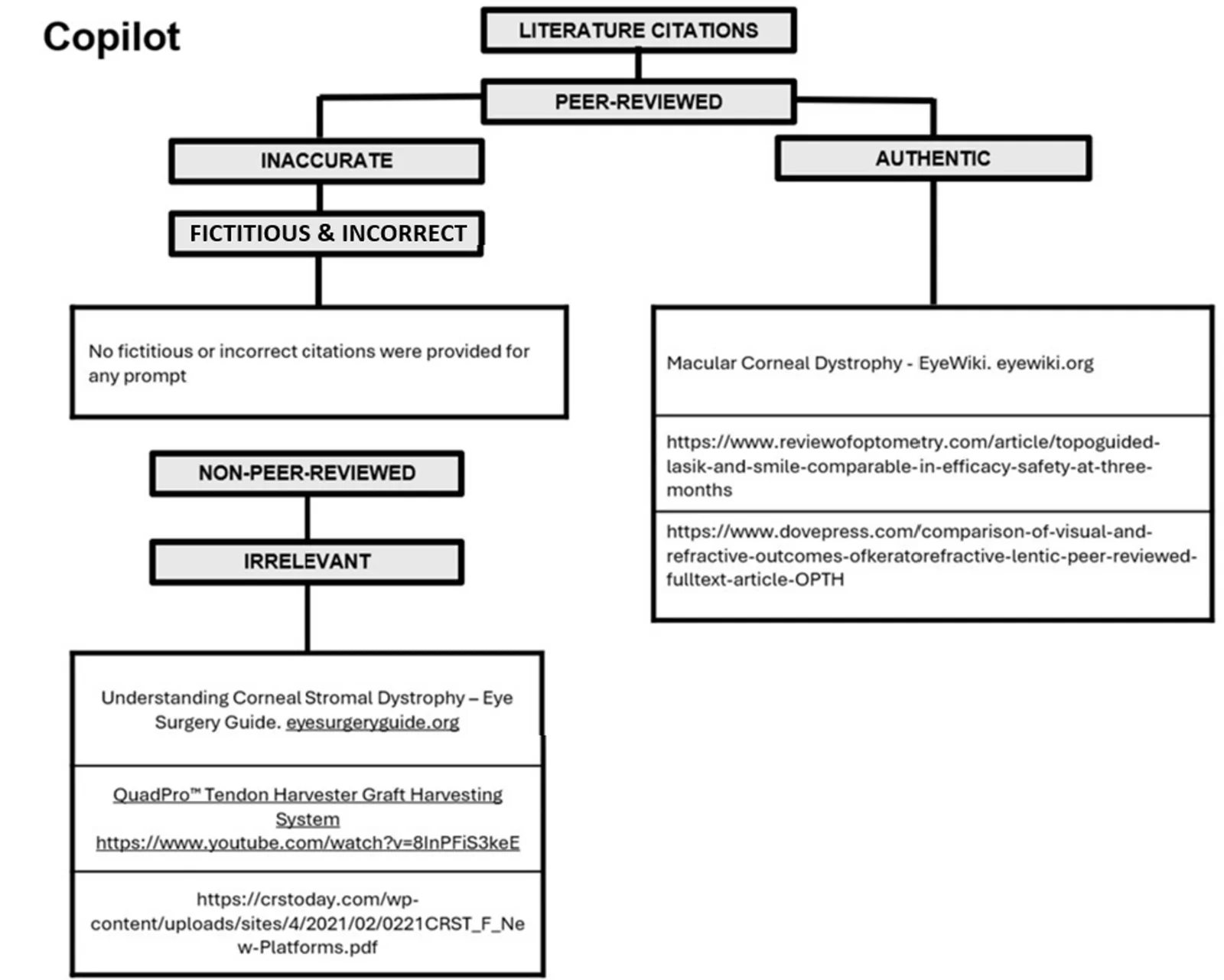
Compilation of provided references from Copilot, categorized as fictitious and incorrect, authentic, and non-peer-reviewed (irrelevant).

**Figure 11:**
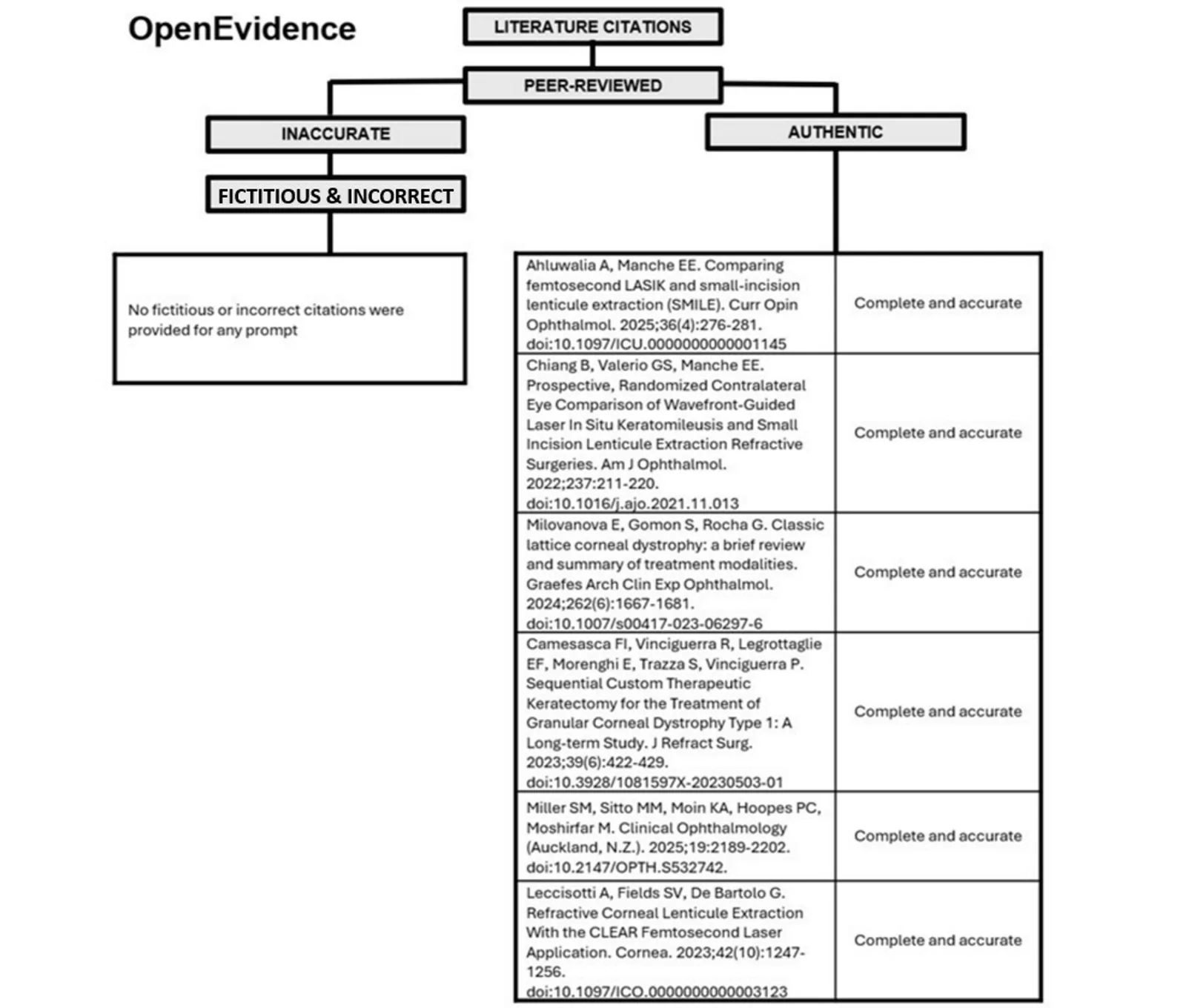
Compilation of provided references from OpenEvidence, categorized as authentic.

## Appendices

Appendix A

**Table 3:** Variable portion of prompt given to LLM models in each ophthalmologic subspecialty. LLM: large language model

Topic	Prompt
1: Stromal dystrophies	a. Looking at the different types of stromal dystrophies including granular, lattice, macular, and reis-buckler what are the conservative measures of treatment and at what point do we progress to surgical interventions? Which surgical modality is the best option for each of the different dystrophies I mentioned above? Make sure to cover each stromal dystrophy I mentioned.
	b. What is the recurrence timeline for each of the four dystrophies in terms of months/years, and are there any differences in the patterns? Additionally, what are the recurrence timelines in months/years for the individual surgical interventions for these dystrophies: PKP, PTK, and DALK? I would like to see the timeline trends and how each differs.
	c. What is the recurrence rate for each of the four stromal dystrophies? When looking at each stromal dystrophy with PTK, PKP, and DALK, how common is the recurrence of the dystrophy after each has been treated with these surgical interventions?
	d. What are the average visual acuities for each of the four stromal dystrophies at the time of surgical treatment with PTK, PKP, and DALK, and post-intervention? Additionally, what is the average improvement in visual acuities after surgical intervention?
	e. What is the typical age for such patients at the time of surgical interventions such as PKP, PTK, and DALK?
2: Corneal transplantation	a. Differentiate the types of bowman layer transplant graft harvesting methods, including manual bowman layer graft harvesting and femtosecond bowman layer graft harvesting. In particular, what are the differences in expected graft thickness? How much leftover stroma would you expect on the graft for each? What are the reasons why a surgeon might select manual over femtosecond harvesting? What indicates the sufficient thickness of the graft? Make sure to answer each question in part A.
	b. Differentiate the surgical techniques, including details on surgical steps of manual, femtosecond, inlay, and onlay bowman layer transplantation. Why might a surgeon decide to perform an onlay instead of an inlay? What are the relative contraindications of each surgical technique? Which is considered less invasive and why? Make sure to answer each question in part B.
	c. What are the different diagnoses/indications for the inlay bowman layer transplantation? What are the different indications for the onlay bowman layer transplantation? What properties of bowman’s layer make it effective in treating keratoconus? What is the risk of graft rejection in bowman layer transplantation? Make sure to answer each question in part C.
	d. What are common preoperative complications related to bowman layer transplantation? What are common intraoperative complications for bowman layer transplantation? What are common postoperative complications noted in the literature for bowman layer transplantation? What are common postoperative patient outcomes for bowman layer transplantation? Would you expect bowman layer transplantation to directly improve a patient's visual acuity in an advanced keratoconus eye? Make sure to answer each question in part D.
3: Keratolenticule Extraction (KLEx) corneal refractive surgery	I want you to provide information on the different kerato-lenticule extraction (KLEx) platforms, specifically SmartSight, CLEAR, SILK, and SMILE (both VisuMax 500 and 800). For these platforms, I want you to provide the following for each: (a-c)
	a. Differentiate the laser settings between each platform, specifically I want to know what the pulse energy, pulse frequency, spot and track separation, laser cut time, lenticule shape, and if the system features centration and cyclotorsion control. Do not forget to report and compare each of the 4 platforms, including the specific SMILE femtosecond lasers.
	b. Differentiate the different surgical parameters, specifically I want to know what the cap thickness and optical zones are for each platform. Make sure you report the findings for each of the four platforms.
	c. Differentiate the different visual outcomes, specifically I want to know at one year, which platform demonstrated the highest percentage of eyes achieving 20/20? Additionally, at six months, which platform demonstrated the highest percentage of eyes with refractive astigmatism within ± 0.50 D?
4: Topography-guided refractive surgery	a. Please comprehensively compare the postoperative visual outcomes of topography-guided LASIK (TG-LASIK) and small incision lenticule extraction (SMILE), including the rate of visual recovery and cumulative uncorrected distance visual acuity (UDVA) up to 3 months.
	b. Comprehensively compare the safety, efficacy, predictability, and stability between TG-LASIK and SMILE. Please include supportive evidence regarding the gain or loss of one or more lines of visual acuity, safety index, efficacy index, vector analysis, and astigmatic accuracy.
	c. Describe the differences in patient-reported outcomes between TG-LASIK and SMILE, such as dry eye issues, glare, halos, and starbursts.
	d. What are the differences in higher-order aberrations, such as trends in horizontal/vertical coma, spherical aberration, and horizontal/oblique trefoil between TG-LASIK and SMILE up to 3 months postoperatively?

Appendix B

Appendix C

Appendix D

Appendix E

Appendix F

Appendix G

Appendix H

**Table 4:** Sampling of unimpressive and inaccurate quotes from each LLM. AI: artificial intelligence; LLM: large language model

Quote	AI model
"SmartSight: At 12 months, 96% of eye achieved UDVA of 20/20 or better."	ChatGPT
"Preferred Surgical Modality: PTK can be utilized for anterior stromal lesions; however, recurrences are common. DALK is often preferred over PKP."	
"Expected Graft Thickness: Femtosecond laser harvesting allows for more precise and predictable graft thickness, typically ranging from 10 to 15 μm with greater uniformity across the graft."	Gemini
"CLEAR & SILK: Specific laser settings like pulse energy and frequency are not consistently detailed in the available literature."	
"Lenticule Shape: All platforms create a plano-convex lenticule, but SmartSight and CLEAR offer more customizable profiles."	OpenEvidence
"Highest % 20/20 at 1 Year: SMILE (VisuMax 800) demonstrated the highest percentage of eyes achieving 20/20 vision at 1 year (93.2% at 6 months, with similar or better outcomes at 1 year extrapolated from large studies)."	
"SMILE shows a higher induction of spherical aberration than TG-LASIK."	Copilot
"Granular Dystrophy: Recurrence rates are approximately 30-50% for PTK and 10-20% for PKP."	
"The VisuMax 800 demonstrated the highest percentage of eyes achieving 20/20 vision at one year, with 96.7% of eyes reaching this benchmark."	
"Inlay is considered the least invasive because it preserves the anterior corneal surface, reduces epithelial disruption, and avoids sutures, minimizing postoperative inflammation and recovery time."	Grok
"Recurrence Timelines for Each Dystrophy: MCD: 10–30 years; mucopolysaccharide deposits recur slowly, starting peripherally and moving centrally."	
"Visual Outcomes: SILK leads in 20/20 attainment (94%) and astigmatism control (91% ≤ ±0.50 D)."	DeepSeek
"Onlay preferred in advanced ectasia where stromal thinning precludes pocket creation."	

Appendix I

**Table 5:** Sampling of impressive and accurate quotes from each LLM. LLM: large language model; AI: artificial intelligence

Quote	AI model
"…femtosecond laser-assisted harvesting offers precision and smoother surfaces but at the expense of increased graft thickness."	ChatGPT
"Lattice Dystrophy: Recurrence post-PKP has a median time of 8.4 years."	
"Surgeons may prefer manual harvesting to obtain thinner grafts, which could be advantageous for specific surgical applications. Conversely, femtosecond laser-assisted harvesting offers precision and smoother surfaces but at the expense of increased graft thickness."	
"Recipient cornea is marked with a trephine of approximately 7-8 mm diameter."	Gemini
"The femtosecond laser-harvested Bowman layer graft is carefully inserted into the laser-created pocket and unfolded."	
"SILK boasts a very rapid laser cut time."	
"VisuMax 500 operates at 500KHz; VisuMax 800 at 2MHz, enabling faster lenticule creation."	OpenEvidence
"TG-LASIK demonstrates a lower rate of loss of two or more lines of Snellen acuity compared to SMILE, with some studies reporting no such losses in TG-LASIK and up to 6.6% in SMILE at 6 months."	
"The VisuMax 500 is the original femtosecond laser platform for SMILE, while the VisuMax 800 introduces enhancements such as faster cutting times and advanced cyclotorsion compensation."	Copilot
"Lattice Dystrophy: Recurrence is observed within 3-7 years for PTK and 5-10 years for PKP."	
"Granular Dystrophy: Surgery is typically performed between ages 40-60."	
Regarding BIOMECHANICAL STRENGTH: "The BL provides tensile strength and rigidity, stabilizing the cornea and halting ectatic progression."	Grok
Regarding CLEAR: "Pulse Frequency: >5 MHz, offering one of the highest repetition rates among KLEx platforms."	
Regarding Manual Harvesting: "Typically yields grafts with a thickness of 10–30 µm, depending on surgeon expertise and dissection technique."	DeepSeek
"A graft thickness of ≥10 µm is considered sufficient for structural support."	
